# High risk mammographic parenchymal patterns and diet: a case–control study

**DOI:** 10.1054/bjoc.2000.1151

**Published:** 2000-06-02

**Authors:** E Sala, R Warren, S Duffy, A Welch, R Luben, N Day

**Affiliations:** Department of Public Health and Primary Care, Strangeways Research Laboratory, Worts Causeway, Cambridge, CB1 8RN, UK; Cambridge and Huntingdon Breast Screening Service, Rosie Maternity Hospital, Robinson Way, Cambridge, CB2 2SW, UK; MRC-Biostatistics Unit, Institute of Public Health, Robinson Way, Cambridge, CB2 2SR, UK

**Keywords:** mammographic parenchymal patterns, diet, tertile

## Abstract

Mammographic parenchymal patterns are related to breast cancer risk and are also thought to be affected by diet. We designed a case–control study comprising 200 cases with high-risk (P2 and DY) mammographic parenchymal pattern and 200 controls with low-risk (N1 and P1) patterns in order to investigate the effect of food and nutrient intake on mammographic patterns. Mammograms were evaluated according to the Wolfe classification system. Dietary data were obtained from 7-day food diaries. Mean daily intake of nutrients was computed from standard UK food tables. The adjusted odds ratio (OR) of having a high-risk pattern in women in the highest tertile of total protein and carbohydrate intake was twice that of women in the lowest tertile (OR = 2.00; 95% confidence interval (CI) 1.06–3.77;*P* = 0.04 and OR = 1.93; 95% CI 1.03–3.59;*P* = 0.04 respectively). There was no excess risk for fat intake. In addition, there was no association between intake of vitamins and mammographic parenchymal patterns. Total meat intake was strongly and positively associated with high-risk patterns among post-menopausal women (OR = 2.50, 95% CI 1.09–5.69, *P* = 0.03). Our study suggests that certain macronutrients and foods such as protein, carbohydrate and meat intake influence the risk of breast cancer through their effects on breast tissue morphology, whereas fat and vitamins do not affect mammographic density. It seems that parenchymal pattern acts as an informative biomarker of the effect of some macronutrient and foodstuffs intake on breast cancer risk. © 2000 Cancer Research Campaign


British Journal of Cancer (2000) **83**(1), 121–126
© 2000 Cancer Research Campaign
doi: 10.1054/ bjoc.2000.1151, available online at http://www.idealibrary.com on IDEAL®

### High risk mammographic parenchymal patterns and diet: a case–control study

**E Sala[1], R Warren[2], S Duffy[3], A Welch[4], R Luben[1] and N Day[1]**

[1]Department of Public Health and Primary Care, Strangeways Research Laboratory, Worts Causeway, Cambridge CB1 8RN, UK; [2]Cambridge and Huntingdon Breast Screening Service, Rosie Maternity Hospital, Robinson Way, Cambridge CB2 2SW, UK; [3]MRC-Biostatistics Unit, Institute of Public Health, Robinson Way, Cambridge CB2 2SR, UK

**Summary** Mammographic parenchymal patterns are related to breast cancer risk and are also thought to be affected by diet. We designed a case–control study comprising 200 cases with high-risk (P2 and DY) mammographic parenchymal pattern and 200 controls with low-risk (N1 and P1) patterns in order to investigate the effect of food and nutrient intake on mammographic patterns. Mammograms were evaluated according to the Wolfe classification system. Dietary data were obtained from 7-day food diaries. Mean daily intake of nutrients was computed from standard UK food tables. The adjusted odds ratio (OR) of having a high-risk pattern in women in the highest tertile of total protein and carbohydrate intake was twice that of women in the lowest tertile (OR = 2.00; 95% confidence interval (CI) 1.06–3.77; $P = 0.04$ and OR = 1.93; 95% CI 1.03–3.59; $P = 0.04$ respectively). There was no excess risk for fat intake. In addition, there was no association between intake of vitamins and mammographic parenchymal patterns. Total meat intake was strongly and positively associated with high-risk patterns among post-menopausal women (OR = 2.50, 95% CI 1.09–5.69, $P = 0.03$). Our study suggests that certain macronutrients and foods such as protein, carbohydrate and meat intake influence the risk of breast cancer through their effects on breast tissue morphology, whereas fat and vitamins do not affect mammographic density. It seems that parenchymal pattern acts as an informative biomarker of the effect of some macronutrient and foodstuffs intake on breast cancer risk. © 2000 Cancer Research Campaign

**Keywords**: mammographic parenchymal patterns; diet; tertile

Wolfe described four different mammographic patterns related to variations in the relative amounts of fat, epithelial and connective tissue in the breast: N1, P1, P2 and DY (Wolfe, 1976). Women with either P2 or DY patterns are considered at greater risk of breast cancer than those with N1 or P1 patterns (Wolfe, 1976; Saftlas and Szklo, 1987; Warner et al, 1992; Sala et al, 1998).

Few studies have assessed the influence of diet on mammographic parenchymal patterns (Brisson et al, 1989; Nordevang et al, 1993). Fat intake was found to be associated with high-risk patterns in these studies, while increased fibre and carotenoid intake were associated with a reduction of high-risk patterns. A clinical trial designed to evaluate the effect of low-fat high-carbohydrate diet found that mammographic dysplasia was significantly associated with high levels of high-density lipoprotein (HDL)-cholesterol and alcohol intake (Boyd et al, 1988, 1989). The Canadian Diet and Breast Cancer Prevention Study showed that, after 2 years of low-fat high carbohydrate, the area of mammographic density was found to be significantly reduced among women with high mammographic density at baseline (Boyd et al, 1998).

The aim of this case–control study nested within the European Prospective Investigation on Cancer in Norfolk study (EPIC–Norfolk) (Day et al, 1999) is to evaluate whether mammographic parenchymal patterns in women without breast cancer are affected by food and nutrient intake.

*Received 17 December 1999*
*Revised 11 February 2000*
*Accepted 11 February 2000*

*Correspondence to:* E Sala

### MATERIALS AND METHODS

### Study population

Study members were women participating in the EPIC-Norfolk study (Day et al, 1999) who were born between February 1921 and December 1946 and who attended the prevalence screening round at the Norwich Breast Screening Programme between November 1989 and December 1997. In addition, they must have been free of breast cancer prior to and at the time of their prevalent screen. A case–control study was designed within the above cohort.

### The case–control study

A total of 9484 women were identified by linking databases from EPIC-Norfolk and the National Health Service (NHS) Regional Breast Screening Programme for Norwich. We aimed to recruit 200 cases with high-risk (P2 and DY) patterns and 200 controls with low-risk (N1 and P1) patterns, matched for age and date of screening. Of these 9484 women identified by linking the databases, 8001 had completed food diaries and 445 of these diaries had been already entered into the EPIC-Norfolk database. A woman was excluded from the total study population if she was diagnosed with a histologically verified breast cancer prior to or at the prevalent screen, if she did not respond to the screening invitations, or after an extensive search, her screening mammogram or screening records were not located. Women who had breast implants were also excluded since it makes the reading of mammographic pattern difficult. We excluded 45 women on the above criteria.

Cases were defined as women from EPIC with a P2/DY Wolfe's mammographic parenchymal pattern on the prevalence screen mammogram who had been diagnosed as normal at that screen. In order for a case to be eligible, a mammogram had to be classified as P2/DY for both sides and both views by the two readers. The two readers worked separately, each blind to the classification of the other readers. There was inter-reader disagreement for 17 women so these were excluded as potential cases. This left 383 women who satisfied the study criteria, i.e. were classified as either NI/P1 or P2/DY patterns. From these, a total of 203 women with P2/DY mammographic patterns according to Wolfe's classification were identified as cases.

For each case, we wished to select one control with an N1/P1 Wolfe's mammographic parenchymal pattern at the prevalence screen mammogram who had been diagnosed as normal at that screen, matched to the case by date of birth (within 1 year) and date of prevalence screen (within 3 months). In order to be eligible, a control mammogram had to be classified as N1/P1 on both sides and both views by the two readers. The readers disagreed for 13 women who were excluded as potential controls. The remaining 62 controls were identified among 8001 women with completed food diaries not yet entered on the database using the same criteria. We randomly selected 248 women that satisfied the inclusion criteria and were matched for date of birth and date of screening with the remaining 62 cases. We then read the mammograms to determine the parenchymal pattern and selected as controls the first 62 women with N1P1 mammographic pattern on both sides and both views that were also individually matched to 62 remaining cases. Their diaries were entered afterwards. Additional information is presented elsewhere (Sala et al, 1999). As a result, 203 cases and 203 individually matched controls remained in the study.

### Risk factor data

Dietary data were obtained from a 7-day food diary given out at the first EPIC health check. Subjects were given instructions on diary completion and returned the diaries by post. Photographs, household measures and individual units were used to help estimate portion size. Mean daily intake of nutrients was computed from the UK standard food tables (Holland et al, 1991). In addition to dietary data, considerable information on menstrual and reproductive history, height and weight, cigarette smoking, hysterectomy and use of exogenous hormones such as hormone replacement therapy (HRT) was available from the EPIC–Norfolk Health and Lifestyle Questionnaire.

### Statistical analysis

Statistical analysis was by conditional logistic regression, which takes into account the matching of controls to cases and produces odds ratio (OR) estimates of relative risk with associated 95% confidence intervals (CI) on these (Breslow and Day, 1980). Descriptive tables complemented the results of these analyses. ORs were adjusted for those variables that were previously found to be associated with high-risk mammographic parenchymal patterns (Sala et al, 1999). Analyses of the association of dietary intake with mammographic parenchymal patterns were repeated adjusted for total energy intake. The *t*-test was used to compare the means of different nutrients and foods.

**Table 1** Characteristics of study population (non-dietary variables)

| Characteristics | Cases (P2+DY) *n* = 203 | Controls (N1+P1) *n* = 203 |
|---|---|---|
| Mean age (years) | 59 | 59 |
| Mean BMI | 25 | 27 |
| Number of children | | |
| 0 | 31 | 16 |
| 1 | 27 | 26 |
| 2 | 96 | 82 |
| 3 | 34 | 47 |
| 4+ | 15 | 29 |
| Unknown | – | 3 |
| Menopausal status | | |
| Premenopausal | 44 | 24 |
| Post-menopausal | 148 | 165 |
| Unknown | 11 | 14 |
| HRT use | | |
| Never | 106 | 131 |
| Past | 22 | 25 |
| Current | 73 | 43 |
| Unknown | 2 | 4 |
| Smoking | | |
| Never | 121 | 120 |
| Past | 62 | 53 |
| Current | 20 | 30 |
| Hysterectomy | | |
| No | 141 | 152 |
| Yes | 60 | 49 |
| Unknown | 2 | 2 |

### RESULTS

Characteristics of the cases and controls (non-dietary variables) are presented in Table 1. The mean ages of cases and controls were similar. Cases had higher BMI than controls. More cases were nulliparous; a similar proportion of cases and controls had between one and three births, and a larger proportion of controls had in excess of three births. Larger proportions of cases were premenopausal, current HRT users and had had a hysterectomy, whereas controls were more likely to be current smokers. Results regarding the HRT use are subject of another paper (Sala et al., 2000).

Table 2 shows the mean daily intakes of dietary macronutrients, micronutrients and foodstuffs among cases and controls. Cases had significantly higher means intakes of energy, carbohydrate, protein, and cereals and bread.

Table 3 shows the unadjusted and adjusted odds ratio (OR) estimates for Wolfe's high-risk mammographic parenchymal patterns and macronutrient intake. Women who were in the highest tertile of energy intake were at greater risk of having a high-risk mammographic pattern compared with those in the lowest tertile (OR = 1.79; 95% CI 1.09–2.91; $P$ = 0.02). We performed the analysis on post-menopausal women separately (data not shown). In this group of women, those who were in the highest tertile of energy intake were twice as likely to have a high-risk mammographic pattern compared with those in the lowest tertile (OR = 2.27; 95% CI 1.20–4.26; $P$ = 0.01). The above findings lost their significance when BMI was included in the model.

The adjusted OR of having a high-risk pattern for women in the highest tertile of total protein intake was twice that of women in the lowest tertile (OR = 2.00; 95% CI 1.06–3.77). A significant trend across the tertiles of protein intake was observed ($P$ = 0.04).

 © 2000 Cancer Research Campaign

**Table 2** Characteristics of study population (dietary variables)

| Characteristics | Mean (s.d.) | | *P*-value |
|---|---|---|---|
| | Cases (P2+DY) *n* = 203 | Controls (N1+P1) *n* = 203 | |
| Total energy (MJ) | 7.0 (1.4) | 6.7 (1.5) | 0.02 |
| Total fat (g) | 62.1 (18.1) | 59.9 (19.9) | 0.2 |
| Protein (g) | 65.3 (12.5) | 62.8 (13.4) | 0.04 |
| Fibre (g) | 14.0 (4.4) | 13.6 (4.2) | 0.3 |
| Carbohydrate (g) | 215.8 (49.6) | 202.9 (46.5) | 0.006 |
| Total carotene (μg) | 1854.9 (1082.1) | 1896.7 (1540.6) | 0.8 |
| Vitamin A (μg) | 538.4 (945.5) | 481.5 (810.7) | 0.5 |
| Vitamin C (mg) | 91.0 (47.6) | 87.2 (47.8) | 0.4 |
| Vitamin E (mg) | 6.5 (2.6) | 6.4 (2.6) | 0.5 |
| Vegetables (g) | 103.3 (62.6) | 95.0 (62.6) | 0.2 |
| Fruits (g) | 178.4 (118.5) | 177.2 (138.8) | 0.9 |
| Cereals and bread (g) | 116.2 (46.9) | 107.2 (44.0) | 0.05 |
| Red meat | 41.3 (36.9) | 38.2 (36.4) | 0.3 |
| White meat | 28.7 (30.1) | 26.9 (27.1) | 0.5 |
| Total meat | 94.2 (47.9) | 88.4 (47.2) | 0.2 |
| Milk (g) | 204.0 (133.0) | 184.1 (139.4) | 0.1 |
| Dairy products (g) | 278.8 (143.8) | 255.1 (155.5) | 0.1 |
| Fish (g) | 33.8 (29.2) | 34.4 (24.4) | 0.8 |
| Alcohol (g) | 7.4 (11.1) | 6.7 (10.0) | 0.5 |

**Table 3** Odds ratio estimates for high-risk mammographic patterns according to dietary macronutrients

| Dietary macronutrients (in tertiles) | Cases (P2+DY) | Controls (N1+P1) | OR | 95% CI | Trend test | OR[a] | 95% CI[a] | Trend test[a] | OR[b] | 95% CI[b] | Trend test[b] |
|---|---|---|---|---|---|---|---|---|---|---|---|
| Total energy (MJ) | | | | | | | | | | | |
| 1 (2.4–6.2) | 57 | 79 | 1.00 | – | 0.02 | 1.00 | – | 0.32 | | | |
| 2 (6.3–7.4) | 70 | 65 | 1.53 | 0.92–2.54 | | 1.35 | 0.70–2.57 | | | | |
| 3 (7.5–11.6) | 76 | 59 | 1.79 | 1.09–2.91 | | 1.38 | 0.74–2.58 | | | | |
| Total fat (g) | | | | | | | | | | | |
| 1 (13.7–52.9) | 64 | 72 | 1.00 | – | 0.36 | 1.00 | – | 0.64 | 1.00 | – | 0.69 |
| 2 (53–68.1) | 68 | 67 | 1.13 | 0.70–1.81 | | 1.08 | 0.59–1.94 | | 0.92 | 0.48–1.76 | |
| 3 (68.2–133.3) | 71 | 64 | 1.24 | 0.77–2.00 | | 1.12 | 0.62–2.02 | | 0.80 | 0.33–1.94 | |
| Total protein (g) | | | | | | | | | | | |
| 1 (27.9–57.9) | 58 | 78 | 1.00 | – | 0.02 | 1.00 | – | 0.004 | 1.00 | – | 0.06 |
| 2 (58–69.1) | 69 | 66 | 1.42 | 0.86–2.35 | | 1.34 | 0.70–2.54 | | 1.41 | 0.70–2.81 | |
| 3 (69.2–104.2) | 76 | 59 | 1.83 | 1.09–3.06 | | 2.00 | 1.06–3.77 | | 2.30 | 1.03–5.16 | |
| Fibre (g) | | | | | | | | | | | |
| 1 (3.7–11.7) | 67 | 69 | 1.00 | – | 0.76 | 1.00 | – | 0.29 | 1.00 | – | 0.39 |
| 2 (11.8–15) | 67 | 68 | 1.01 | 0.63–1.61 | | 1.06 | 0.60–1.89 | | 1.04 | 0.59–1.87 | |
| 3 (15.1–28.3) | 69 | 66 | 1.08 | 0.66–1.74 | | 1.40 | 0.77–2.53 | | 1.34 | 0.72–2.46 | |
| Carbohydrate (g) | | | | | | | | | | | |
| 1 (77.7–186.2) | 55 | 81 | 1.00 | – | 0.01 | 1.00 | – | 0.04 | 1.00 | – | 0.06 |
| 2 (186.3–231.2) | 72 | 63 | 1.66 | 1.02–2.69 | | 1.67 | 0.91–3.05 | | 1.89 | 0.90–3.92 | |
| 3 (231.3–382.1) | 76 | 59 | 1.88 | 1.15–3.06 | | 1.93 | 1.03–3.59 | | 2.50 | 1.00–6.24 | |

[a]Adjusted for menopausal status, parity, HRT, BMI. [b]Adjusted for menopausal status, parity, HRT, BMI, total energy intake.

The above findings persisted when the analysis were limited to post-menopausal women only (OR = 2.20; 95% CI 1.04–4.63; $P$ = 0.03). The adjusted OR of having a high-risk pattern for women in the highest tertile of carbohydrate intake was almost twice that of women in the lowest tertile (OR = 1.93; 95% CI 1.03–3.59). A significant trend across the tertiles of carbohydrate intake was observed ($P$ = 0.04). Similar results were obtained among post-menopausal women (OR = 2.22; 95% CI 1.02–4.79; $P$ = 0.02). Adjusting for total energy intake resulted in an increase in the ORs but, since both protein and carbohydrate were highly correlated (correlation coefficients were 0.74 and 0.82 respectively) with total energy intake, the CI became wider and significance became borderline. There was no association between mammographic patterns and intakes of fat and fibre.

Table 4 shows the unadjusted and adjusted OR estimates for Wolfe's high-risk mammographic parenchymal patterns according to micronutrients (vitamins). There was no association between mammographic patterns and vitamin intake.

Table 5 shows the unadjusted and adjusted OR estimates for Wolfe's high-risk mammographic parenchymal patterns and foodstuffs intake. Relative to women in the lowest tertile of total meat intake, the OR of having a high-risk mammographic pattern for

*© 2000 Cancer Research Campaign* 

**Table 4** Odds ratio estimates for high-risk mammographic patterns according to micronutrients

| Dietary vitamins (in tertiles) | Cases (P2+DY) | Controls (N1+P1) | OR | 95% CI | Trend test | OR[a] | 95% CI[a] | Trend test[a] | OR[b] | 95% CI[b] | Trend test[b] |
|---|---|---|---|---|---|---|---|---|---|---|---|
| Total carotene (µg) | | | | | | | | | | | |
| 1 (198.4–1227.2) | 63 | 73 | 1.00 | – | 0.39 | 1.00 | – | 0.27 | 1.00 | – | 0.31 |
| 2 (1227.3–2118.1) | 71 | 64 | 1.31 | 0.80–2.16 | | 1.68 | 0.90–3.12 | | 1.67 | 0.89–3.12 | |
| 3 (2118.2–15670.5) | 69 | 66 | 1.25 | 0.74–2.10 | | 1.43 | 0.75–2.71 | | 1.38 | 0.72–2.63 | |
| Vitamin A (µg) | | | | | | | | | | | |
| 1 (16.3–199.9) | 62 | 74 | 1.00 | – | 0.44 | 1.00 | – | 0.76 | 1.00 | – | 0.89 |
| 2 (200–348.9) | 73 | 62 | 1.43 | 0.87–2.35 | | 1.46 | 0.78–2.72 | | 1.34 | 0.70–2.56 | |
| 3 (349–7024.3) | 68 | 67 | 1.21 | 0.75–1.94 | | 1.01 | 0.56–1.81 | | 0.90 | 0.48–1.71 | |
| Vitamin C (mg) | | | | | | | | | | | |
| 1 (9.0–62.8) | 68 | 68 | 1.00 | – | 0.35 | 1.00 | – | 0.45 | 1.00 | – | 0.47 |
| 2 (62.9–101.5) | 60 | 75 | 0.82 | 0.51–1.30 | | 1.08 | 0.60–1.93 | | 1.09 | 0.60–1.95 | |
| 3 (101.6–363) | 75 | 60 | 1.31 | 0.79–2.17 | | 1.32 | 0.69–2.51 | | 1.30 | 0.68–2.47 | |
| Vitamin E (mg) | | | | | | | | | | | |
| 1 (1.7–5.1) | 67 | 69 | 1.00 | – | 0.85 | 1.00 | – | 0.62 | 1.00 | – | 0.41 |
| 2 (5.2–7.1) | 68 | 67 | 1.04 | 0.66–1.63 | | 0.95 | 0.54–1.68 | | 0.88 | 0.49–1.58 | |
| 3 (7.2–18.7) | 68 | 67 | 1.05 | 0.63–1.74 | | 0.85 | 0.45–1.61 | | 0.73 | 0.36–1.44 | |

[a] Adjusted for menopausal status, parity, HRT, BMI. [b] Adjusted for menopausal status, parity, HRT, BMI, total energy intake.

**Table 5** Odds ratio estimates for high-risk mammographic patterns according to foodstuffs

| Food stuffs (in tertiles) | Cases (P2+DY) | Controls (N1+P1) | OR | 95% CI | Trend test | OR[a] | 95% CI[a] | Trend test[a] | OR[b] | 95% CI[b] | Trend test[b] |
|---|---|---|---|---|---|---|---|---|---|---|---|
| Vegetables (g) | | | | | | | | | | | |
| 1 (0–64.5) | 64 | 72 | 1.00 | – | 0.27 | 1.00 | – | 0.70 | 1.00 | – | 0.65 |
| 2 (64.6–118.4) | 67 | 68 | 1.13 | 0.70–1.81 | | 1.19 | 0.65–2.15 | | 1.22 | 0.67–2.23 | |
| 3 (118.5–412.5) | 72 | 63 | 1.33 | 0.79–2.22 | | 1.14 | 0.61–2.11 | | 1.18 | 0.63–2.18 | |
| Cereals & bread | | | | | | | | | | | |
| 1 (0–92.1) | 61 | 75 | 1.00 | – | 0.08 | 1.00 | – | 0.21 | 1.00 | – | 0.28 |
| 2 (92.2–124.5) | 67 | 68 | 1.24 | 0.75–2.02 | | 0.96 | 0.53–1.73 | | 0.92 | 0.50–1.67 | |
| 3 (124.6–356.8) | 75 | 60 | 1.55 | 0.95–2.53 | | 1.44 | 0.79–2.62 | | 1.35 | 0.72–2.50 | |
| Fruits (g) | | | | | | | | | | | |
| 1 (0–111.6) | 66 | 70 | 1.00 | – | 0.68 | 1.00 | – | 0.26 | 1.00 | – | 0.25 |
| 2 (111.7–204.4) | 68 | 67 | 1.07 | 0.66–1.73 | | 1.37 | 0.75–2.47 | | 1.33 | 0.73–2.42 | |
| 3 (204.5–936.3) | 69 | 66 | 1.10 | 0.69–1.76 | | 1.43 | 0.78–2.60 | | 1.43 | 0.78–2.60 | |
| Red meat (g) | | | | | | | | | | | |
| 1 (0–17) | 66 | 70 | 1.00 | – | 0.38 | 1.00 | – | 0.46 | 1.00 | – | 0.66 |
| 2 (17.1–49.8) | 65 | 70 | 0.99 | 0.60–1.62 | | 1.10 | 0.58–2.06 | | 1.05 | 0.54–2.01 | |
| 3 (49.9–200) | 72 | 63 | 1.28 | 0.75–2.19 | | 1.29 | 0.65–2.54 | | 1.18 | 0.57–2.41 | |
| White meat (g) | | | | | | | | | | | |
| 1 (0–8.5) | 76 | 65 | 1.00 | – | 0.85 | 1.00 | – | 0.96 | 1.00 | – | 0.98 |
| 2 (8.6–36.7) | 53 | 78 | 0.60 | 0.37–0.98 | | 0.61 | 0.33–1.12 | | 0.61 | 0.33–1.12 | |
| 3 (36.8–162.4) | 74 | 60 | 1.07 | 0.63–1.79 | | 1.00 | 0.53–1.87 | | 1.00 | 0.53–1.88 | |
| Total meat (g) | | | | | | | | | | | |
| 1 (0–70) | 57 | 79 | 1.00 | – | 0.09 | 1.00 | – | 0.17 | 1.00 | – | 0.27 |
| 2 (70.1–105.5) | 76 | 59 | 1.79 | 1.09–2.92 | | 1.64 | 0.90–2.95 | | 1.61 | 0.88–2.93 | |
| 3 (105.6–258.6) | 70 | 65 | 1.53 | 0.92–2.54 | | 1.68 | 0.90–3.11 | | 1.59 | 0.83–3.04 | |
| Milk (g) | | | | | | | | | | | |
| 1 (0–118.5) | 62 | 74 | 1.00 | – | 0.15 | 1.00 | – | 0.46 | 1.00 | – | 0.64 |
| 2 (0–118.6–226.4) | 67 | 68 | 1.17 | 0.73–1.86 | | 1.11 | 0.63–1.93 | | 1.11 | 0.63–1.93 | |
| 3 (226.5–927.6) | 74 | 61 | 1.41 | 0.88–2.23 | | 1.23 | 0.69–2.19 | | 1.16 | 0.69–2.12 | |
| Dairy products (g) | | | | | | | | | | | |
| 1 (0–185.9) | 59 | 77 | 1.00 | – | 0.09 | 1.00 | – | 0.17 | 1.00 | – | 0.26 |
| 2 (186–311.6) | 71 | 64 | 1.49 | 0.90–2.45 | | 1.55 | 0.84–2.85 | | 1.53 | 0.82–2.83 | |
| 3 (311.7–985.1) | 73 | 62 | 1.51 | 0.94–2.40 | | 1.58 | 0.89–2.79 | | 1.52 | 0.83–2.79 | |
| Fish (g) | | | | | | | | | | | |
| 1 (0–21.4) | 72 | 64 | 1.00 | – | 0.50 | 1.00 | – | 0.97 | 1.00 | – | 0.97 |
| 2 (21.5–40) | 65 | 70 | 0.81 | 0.49–1.33 | | 0.93 | 0.49–1.73 | | 0.92 | 0.49–1.71 | |
| 3 (40.1–208.1) | 66 | 69 | 0.84 | 0.51–1.37 | | 0.99 | 0.54–1.78 | | 0.97 | 0.53–1.76 | |
| Alcohol (g) | | | | | | | | | | | |
| Non-drinkers | 68 | 79 | 1.00 | – | 0.25 | 1.00 | – | 0.61 | 1.00 | – | 0.66 |
| Drinkers | 135 | 124 | 1.28 | 0.84–1.95 | | 1.19 | 0.69–2.03 | | 1.16 | 0.67–1.99 | |

[a] Adjusted for menopausal status, parity, HRT, BMI. [b] Adjusted for menopausal status, parity, HRT, BMI and total energy intake.

 *© 2000 Cancer Research Campaign*

**Table 6** Multivariate model results with effect of energy, carbohydrate and protein mutually adjusted

| Dietary nutrients (in tertiles) | OR[a] | 95% CI[a] | Trend test[a] |
|---|---|---|---|
| Total energy (MJ) | | | |
| 1 (2.4–6.2) | 1.00 | – | 0.13 |
| 2 (6.3–7.4) | 0.68 | 0.29–1.57 | |
| 3 (7.5–11.6) | 0.40 | 0.13–1.19 | |
| Carbohydrate (g) | | | |
| 1 (77.7–186.2) | 1.00 | – | 0.06 |
| 2 (186.3–231.2) | 2.04 | 0.95–4.34 | |
| 3 (231.3–382.1) | 2.55 | 0.98–6.59 | |
| Total protein (g) | | | |
| 1 (27.9–57.9) | 1.00 | – | 0.06 |
| 2 (58–69.1) | 1.51 | 0.75–3.03 | |
| 3 (69.2–104.2) | 2.41 | 1.05–5.49 | |

[a]Also adjusted for menopausal status, parity, HRT, BMI.

women in the highest tertile was 1.68 (95% CI 0.90–3.11). Total meat intake was strongly and positively associated with high-risk patterns among post-menopausal women. Relative to the lowest tertile, women in the highest tertile of the total meat intake were significantly more likely to have a high-risk pattern (OR = 2.50, 95% CI 1.09–5.69, $P$ = 0.03). The above findings lost their significance when total energy intake was included in the model.

There was no association between mammographic patterns and intake of red meat, white meat, vegetables, cereals, fruits, milk, dairy products, fish and alcohol.

Clearly, one might expect some positive or negative collinearity among nutrients, particularly energy, carbohydrate and protein. Accordingly, we fitted these in a multivariate model, each adjusted for the other two factors (and other possible confounding variables). Results are shown in Table 6. The positive effect of energy vanishes after adjustment, and the ORs associated with protein and carbohydrate remain similar, an approximate doubling of risk in the two higher tertiles.

To investigate the relationship between mammographic parenchymal patterns and diet further we repeated the analysis considering women with N1 pattern as the lowest risk and those with DY pattern as the highest risk pattern. We found a significant positive association between dietary intakes of total energy, protein, carbohydrate, milk, dairy products, red meat, total meat and DY mammographic parenchymal pattern (compared to N1), and a significant inverse relationship between fish intake and DY pattern.

### DISCUSSION

In this study, we found a strong positive association between intake of certain macronutrients such as protein and carbohydrate and Wolfe's high-risk mammographic parenchymal patterns of breast tissue. There was no excess risk for fat intake. In addition, there was no association between intake of vitamins and mammographic parenchymal patterns. Among post-menopausal women, we found a strong positive relationship between dietary intake of total meat and high-risk parenchymal patterns.

Part of the slowness in recognizing breast density, as a risk factor for breast cancer may be the difficulty of finding a reliable method for assessing the parenchymal patterns. The Wolfe classification system, as well as other methods of classification, depends on percentages of the breast with dense parenchyma implying that an association with breast size is inevitable. It is also inevitable that all methods of classification of breast density are dependent on BMI and WHR since higher BMI and WHR means more fatty tissue generally and more fatty replacement in the breast. Thus, it would be most useful to define a measure of mammographic density independent of body habitus that will estimate the volume (three-dimensional) of the breast tissue which appears dense on the mammogram.

Our study design minimized the potential for bias in our findings. Mammographic parenchymal pattern reading was done without knowledge of dietary and risk factor data avoiding systematic error due to observation bias. In addition, subjects completed dietary diaries with no knowledge of their case or control status. There is a possibility of random misclassification of dietary habits and limitations of food composition tables leading to regression dilution bias and hence to an underestimation of the effect of the true relationship between dietary components and mammographic patterns. Validation studies undertaken in the EPIC–Norfolk cohort have shown that, for many food groups and dietary constituents, 7-day food diaries are superior to Food Frequency Questionnaires (FFQs) (Bingham et al, 1994). Biases in self-reported dietary intakes have been reported to be greater for obese subjects than for lean subjects (Schoeller et al, 1990). This was the case in our study, where women in the highest tertile of BMI reported lower energy intake compared with those in the lowest tertile. Of the main sources of energy, this apparent underreporting was greatest for carbohydrate followed by fat and was least for protein.

The unadjusted results in Table 3 showed increased risk associated with high intake of energy, protein and carbohydrate. There was no effect of fat. After adjustment in a multivariate model, the effect of energy vanished but the ORs for protein and carbohydrate remained similar. This suggests that the increased risk associated with high energy intake is related only to energy from protein and carbohydrates.

The associations observed are unlikely to be explained by the confounding effect of other possible risk factors for high-risk mammographic patterns since these were adjusted for in the analysis. Results of the association between dietary variables with mammographic parenchymal patterns were presented both with and without total energy intake in the models. Adjustment for total energy intake might be unnecessary since total energy intake and intakes of carbohydrate, fat and protein were highly correlated. Adjustment for total energy may reduce the precision of the estimates without increasing the validity. Also, since several of these foods and nutrients are correlated with each other, there may be some mutual confounding.

Few studies have investigated the influence of diet on mammographic parenchymal patterns (Brisson et al., 1989; Nordevang et al, 1993). A case–control study found that saturated fat intake was associated with an increase in high-risk mammographic patterns, while increased fibre and carotenoid intakes were associated with a reduction of high-risk patterns (Brisson et al, 1989). A cross-sectional study found that breast cancer patients with the DY pattern reported significantly higher intakes of total fat, monounsaturated fatty acids, polyunsaturated fatty acids and alpha-tocopherol compared with women with N1 pattern (Nordevang et al, 1993). The Canadian Diet and Breast Cancer Prevention Study showed that, after 2 years of low-fat high carbohydrate, the area of mammographic density was found to be significantly reduced among women who were identified as having high mammographic

*© 2000 Cancer Research Campaign* 

density at baseline (Boyd et al, 1998; Knight et al, 1999). The most significant dietary variable associated with reduction in percentage of density among women going through menopause was reduction of dietary cholesterol intake ($P = 0.001$). However the magnitude of the reduction was small (6.1% and 11% in all women and in those going through menopause respectively) and it is unlikely to be associated with an important reduction in breast cancer risk (Boyd et al, 1998; Knight et al, 1999).

The epidemiological evidence for the role of dietary fat intake in risk of breast cancer is inconclusive (Willett et al, 1992; Boyd et al, 1993; Hunter et al, 1996; Clavel Chapelon et al, 1997). However, there is evidence that high meat intake increases breast cancer risk (Boyd et al, 1993; Toniolo et al, 1994), while fish intake decreases it (Vatten et al, 1990). The mechanisms by which diet could affect breast tissue morphology are still not clear. One mechanism could involve female hormone levels. There is evidence that vegetarian women have lower blood and urine levels of some estrogens than do non-vegetarians (Armstrong et al, 1981; Goldin et al, 1982). They also have low fat intake and high fibre intake. It is unclear which aspect of the diet influences the endogenous hormone levels.

Our study suggests that certain macronutrients and foods such as protein, carbohydrate and meat intake may influence the risk of breast cancer through their effects on breast tissue morphology as assessed by mammography, whereas fat and vitamins do not affect mammographic density. Parenchymal patterns appear to act as an informative biomarker of the effect of some macronutrients and foods intake on breast cancer risk. They may be most useful as means of testing hypothesis about potential preventive strategies.

### ACKNOWLEDGEMENTS

We thank Anglia and Oxford Health Authority, R & D Programme for funding this study. We are most grateful to the staff of EPIC–Norfolk for their contribution to the study. We thank Dr Graham Hurst, director of the Norwich Breast Screening Unit, and all the staff of the Norwich Breast Screening Unit for their invaluable help during data collection. We also thank Dr Jenny McCann for help with data collection and for her comments on the manuscript.

### REFERENCES

Armstrong BK, Brown JB, Clarke HT, Crooke DK, Hahnel R, Masarei JR and Ratajczak T (1981) Diet and reproductive hormones: a study of vegetarian and non-vegetarian postmenopausal women. *J Natl Cancer Inst* **67**: 761–767

Bingham S, Gill C, Welch A, Cassidy A, Khaw K-T, Sneyd MJ, Key TJ, Roe L and Day NE (1994) Comparison of dietary assessment methods in nutritional epidemiology: weighed records vs 24 h recalls, food frequency questionnaires and estimated diet records. *Br J Nutr* **74**: 619–642

Boyd NF, Cousins M, Beaton M, Fishell E, Wright B, Fish E, Kriukov V, Lockwood G, Tritchler D, Hanna W and Page DL (1988) Clinical trial of low-fat, high-carbohydrate diet in subjects with mammographic dysplasia: report of early outcomes. *J Natl Cancer Inst* **80**: 1244–1248

Boyd NF, McGuire V, Fishell E, Kuriov V, Lockwood G and Tritchler D (1989) Plasma lipids in premenopausal women with mammographic dysplasia. *Br J Cancer* **59**: 766–771.

Boyd NF, Martin LJ, Noffel M, Lockwood GA and Tritchler DL (1993) A meta-analysis of studies of dietary fat and breast cancer risk. *Br J Cancer* **68**: 627–636

Boyd NF, Greenberg C, Lockwood G, Little L, Martin L, Byng JW, Yaffe MJ and Tritchler D (1998) Effects of 2 years of low-fat, high-carbohydrate diet on radiologic features of the breast: results from a randomized trial. *J Natl Cancer Inst* **89**: 488–496

Breslow NE and Day NE (1980) *Statistical Methods in Cancer Research*, Vol. 1. IARC Scientific Publications, Lyan

Brisson J, Verreault R, Morrison AS, Tennina S and Meyer F (1989) Diet, mammographic features of breast tissue, and breast cancer risk. *Am J Epidemiol* **130**: 14–24

Clavel Chapelon F, Niravong M and Joseph RR (1997) Diet and breast cancer: review of the epidemiologic literature. *Cancer Detect Prev* **21**: 426–440

Day NE, Oakes S, Luben R, Khaw K.-T, Bingham S, Welch A and Wareham N (1999) Epic in Norfolk: study design and characteristics of the cohort. *Br J Cancer* **80**: 95–103

Goldin BR, Adlercreutz H, Gorbach SL, Warram JH, Dwyer JT, Swenson L and Woods MN (1982) Estrogen excretion patterns and plasma levels in vegetarian and omnivorous women. *N Engl J Med* **307**: 1542–1547

Holland B, Welch AA, Unwin ID, Buss DH, Paul AA and Southgate DAT (1991) *McCance and Widdowson's The Composition of Foods*, 5th edn. The Royal Society of Chemistry, Cambridge.

Hunter DJ, Spiegelman D, Adami HO, Beeson L, van den Brandt PA, Folsom AR and Fraser GE (1996) Cohort studies of fat intake and risk of breast cancer – a pooled analysis. *N Engl J Med* **334**: 356–361

Knight JA, Martin LJ, Greenberg CV, Lockwood GA, Byng JW, Yaffe MJ, Tritchler DL and Boyd NF (1999) Macronutrient intake and change in mammographic density at menopause: results from a randomised trial. *Cancer Epidemiol Biomarkers Prev* **8**: 123–128

Nordevang E, Azavedo E, Svane G, Nilsson B and Holm LE (1993) Dietary habits and mammographic patterns in patients with breast cancer. *Breast Cancer Res Treat* **26**: 207–215

Saftlas AF and Szklo M (1987) Mammographic parenchymal patterns and breast cancer risk. *Epidemiol Rev* **9**: 146–174

Sala E, Warren RML, McCann J, Duffy S, Day N and Luben R (1998) Mammographic parenchymal patterns and mode of detection: implications for the breast screening programme. *J Med Screen* **5**: 207–212

Sala E, Warren RML, McCann J, Duffy S, Luben R and Day N (1999) High-risk mammographic parenchymal patterns and anthropometric measures: a case–control study. *Br J Cancer* **81**: 1257–1261

Sala E, Warren RML, McCann J, Duffy S, Luben R and Day N (2000) High-risk mammographic parenchymal patterns, hormone replacement therapy and other risk factors: a case–control study. *Int J Epidemiol* (in press)

Schoeller DA, Bandini LG and Dietz WH (1990) Innaccuracies in self-reported intake identified by comparison with the doubly labelled water method. *Can J Physiol Pharmacol* **68**: 941–949

Toniolo P, Riboli E, Shore RE and Pasternack BS (1994) Consumption of meat, animal products, protein, and fat and risk of breast cancer: a prospective cohort study in New York. *Epidemiology* **5**: 391–397

Vatten LJ, Solvoll K and Loken EB (1990) Frequency of meat and fish intake and risk of breast cancer in a prospective study of 14 500 Norweigian women. *Int J Cancer* **46**: 12–15

Warner E, Lockwood G, Math M, Tritchler D and Boyd NF (1992) The risk of breast cancer associated with mammographic parenchymal patterns: a meta-analysis of the published literature to examine the effect of method of classification. *Cancer Detect Prevent* **16**: 67–72

Willett WC, Hunter DJ, Stampfer MJ, Colditz GA, Manson JE, Spiegelman D, Rosner B, Hennekens CH and Speizer FE (1992) Dietary fat and fibre in relation to risk of breast cancer. An 8-year follow-up. *JAMA* **268**: 2037–2044

Wolfe JN (1976) Breast patterns as an index of risk for developing breast cancer. *Am J Roentgenol* **126**: 1130–1139

*© 2000 Cancer Research Campaign*
